# Klotho inhibits EGF-induced cell migration in Caki-1 cells through inactivation of EGFR and p38 MAPK signaling pathways

**DOI:** 10.18632/oncotarget.25481

**Published:** 2018-06-01

**Authors:** Mehdi Dehghani, Reynolds K. Brobey, Yue Wang, Glauco Souza, Robert J. Amato, Kevin P. Rosenblatt

**Affiliations:** ^1^ Division of Oncology, Department of Internal Medicine, The University of Texas Health Science Center at Houston McGovern Medical School, Houston, Texas 77030, United States of America; ^2^ n3D Biosciences, Inc., Houston, Texas 77030, United States of America; ^3^ NX Prenatal, Inc., Bellaire, Texas 77401, United States of America; ^4^ Consultative Genomics, PLLC, Bellaire, Texas 77401, United States of America

**Keywords:** Klotho, clear-cell renal cell carcinoma, cell migration, wound healing assay, Caki-1

## Abstract

Klotho is a single-pass transmembrane protein with documented anti-cancer properties. Recent reports have implicated Klotho as an inhibitor of transforming growth factor β1 induced cell migration in renal fibrosis. Overexpression of epidermal growth factor receptor (EGFR) is known to promote tumor initiation and progression in clear-cell renal cell carcinoma (cRCC). We tested our hypothesis that Klotho inhibits EGF-mediated cell migration in cRCC by interfering with the EGFR signaling complex and mitogen-activated protein kinase (MAPK) pathways. We performed cell adhesion, migration, and biochemical studies *in vitro* using Caki-1 cell line. In addition, we validated the cell culture studies with expression analysis of six de-identified FFPE tissues from primary and metastatic cRCC patients. Our studies show that Klotho inhibited EGF-induced Caki-1 de-adhesion and decreased spreading on collagen type 1. Klotho also inhibited EGF-induced α2β1 integrin-dependent cell migration on collagen type 1. To test the involvement of MAPK pathways in EGF-induced Caki-1 cell motility, the cells were pretreated with either SB203580, a specific p38 MAPK inhibitor, or Klotho. SB203580 blocked the EGF-induced Caki-1 cell migration. Klotho had a comparable inhibitory effect. Our FFPE clinical specimens revealed decreased Klotho mRNA expression compared to a control, non-cancer kidney tissue. The decrease in Klotho mRNA levels correlated with increased c-Src expression, while E-Cadherin was relatively reduced in metastatic FFPE specimens where Klotho was least expressed. Taken together, these results suggest that secreted Klotho inhibits EGF-induced pro-migratory cell morphological changes and migration in Caki-1 cells. Our data additionally suggest that decreased Klotho expression may be involved in cRCC metastasis.

## INTRODUCTION

Klotho is a type 1 transmembrane protein with homology to β-glucosidase and was discovered as a mutated gene in a mouse showing several phenotypes resembling human aging [1, 2]. Klotho is also highly expressed in renal tubular cells. The approximately 120 *k*Da extracellular domain is released into circulation by ectodomain shedding and acts as a humoral factor [3, 4]. One of the mechanisms of action of Klotho is its suppressive interaction with insulin and insulin-like growth factor–1 receptors (IGF-1R) [5]. This finding supported studies implicating Klotho as a tumor suppressor in breast, gastric, and pancreatic cancers [6–9]. Subsequent data also indicated that Klotho inhibits transforming growth factor β1 (TGFβ1)–induced cell migration in renal fibrosis [3].

Cell migration is a crucial step in cancer invasion and metastasis that requires signaling cooperation between growth factor receptors and cell adhesion molecules [10]. One of the major families of cell adhesion molecules are the integrins, which comprise heterodimers of α and β subunits. The extracellular domain of non-covalently bound α and β subunits together define the specificity of integrins to bind to different extracellular matrix components [10]. Upon engagement with their cognate ligands, integrins undergo conformational changes and their cytoplasmic domains interact with the cytoskeleton through adaptor proteins such as talin, vinculin, and paxillin, as well as with some signaling molecules, notably focal adhesion kinase (FAK) and the oncogene Src; both are activated upon extracellular matrix binding or growth factor stimulation [11]. After integrin clustering, these multiprotein complexes form microscopic structures called focal adhesions [11]. The stability of focal adhesion complexes defines the motile behavior of cells [10, 11]. Growth factors such as epidermal growth factor (EGF) increase cell motility by changing the strength of cell adhesiveness mediated by focal adhesions [12]. In addition, it has been shown that several proteins involved in epidermal growth factor receptor (EGFR) signaling pathways, including mitogen-activated protein kinases (MAPKs), can affect focal adhesion stability and, consequently, cell migration [10, 11, 13]. Overexpression of EGFR is associated with tumor initiation and progression in several solid tumors, including clear-cell renal cell carcinoma (cRCC) [14–16]. Moreover, in the case of cRCC, which has a tendency to disseminate to distance sites, the epithelial-stromal interaction is considered essential, with collagen degradation/re-deposition occurring within the tumor. Subsequent binding with integrins and growth factors may serve an important purpose for migration and invasion [17, 18].

Because Klotho, a tumor suppressor, has inhibitory effects on growth factor receptors such as the fibroblast growth factor receptor (FGFR), IGF-1R, and TGFβ1R [6–8], we hypothesized that Klotho would mitigate EGF-induced cell migration in cRCC. For this purpose, we used a Caki-1 cell model of cRCC and conducted *in vitro* cell migration and biochemical studies in the absence or presence of secreted Klotho. In addition, we describe an expression analysis of three primary and three metastatic FFPE clinical cRCC specimens targeting selected markers associated with epithelial to mesenchymal transition (EMT) and cell migration.

## RESULTS AND DISCUSSION

### Klotho's inhibition of EGF-induced p38 MAPK phosphorylation coincides with impaired Caki-1 cell migration on collagen type 1

**Figure 1:**
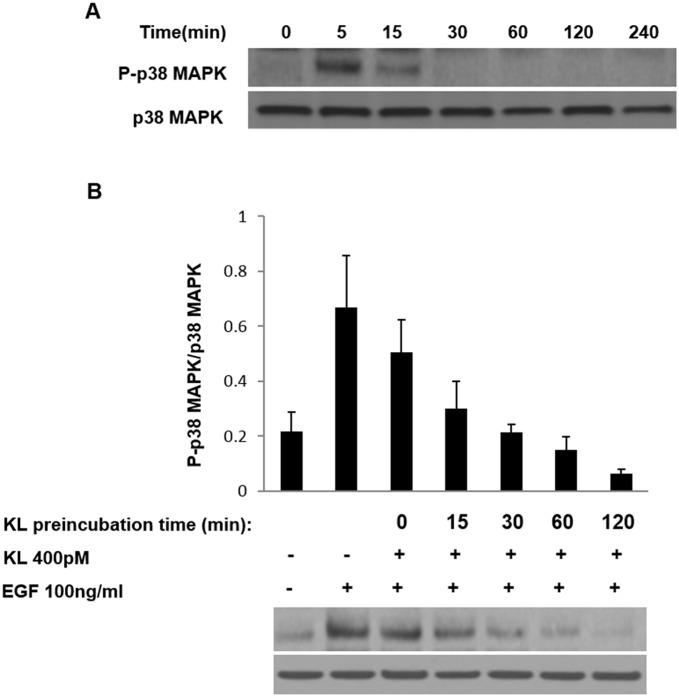
Klotho inhibits EGF-induced p38 MAPK phosphorylation in Caki-1 cells **(A)** Cells were pretreated with EGF (100ng/ml) and harvested after the indicated times. Lysates were collected, and p38 MAPK phosphorylation levels were analyzed by Western blotting using the antiphospho-p38 antibody. The same membrane was striped and re-probed with a different antibody for measuring total-p38 MAPK protein levels. **(B)** Caki-1 cells were preincubated with 400pM of the secreted form of the Klotho (KL) protein at the indicated times followed by EGF (100ng/ml) stimulation for additional 5 min. Cell lysates were subjected to Western blot analysis as described in (A). Plots indicate means ± S.E.M of three independent experiments.

**Figure 2:**
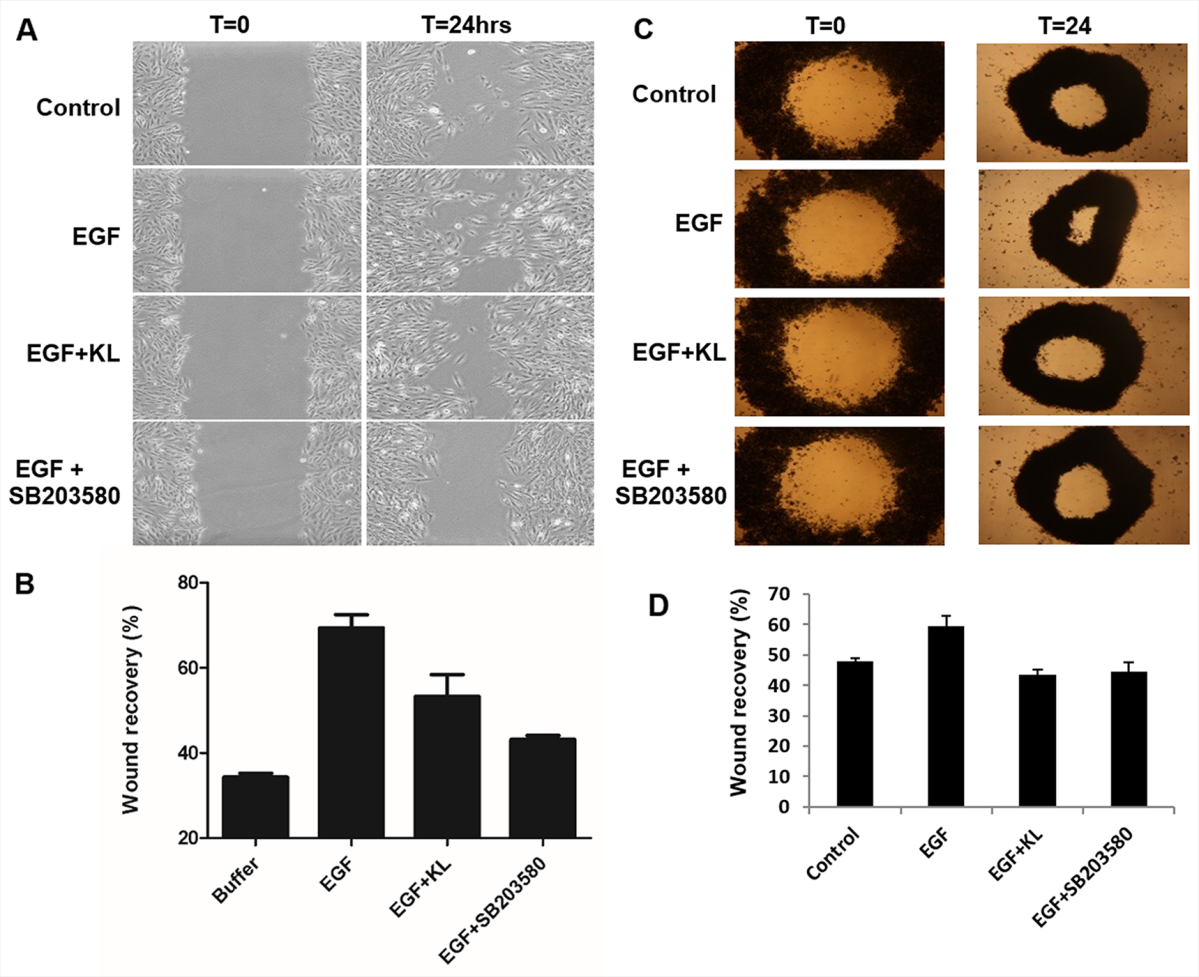
Klotho inhibits Caki-1 cell migration on collagen type 1 **(A)** Classical *in vitro* wound healing assay was performed using near-confluent serum-starved Caki-1 cells grown on collagen type 1. Cells were either pretreated with 400pM Klotho (KL) or 1μM p38 MAPK-specific inhibitor SB203580 as positive control, for 60 min followed by EGF (100ng/ml) treatment. Cell culture images shown here were taken at 0 and 24 h. **(B)** Plots of quantification of the resultant cell motility values were computed by gap surface area measurements for four selected microscopic fields in each assay condition. The degree of migration is expressed as % wound closure compared with zero time point. The results represent means ± S.E.M of three independent experiments. **(C)** Wound healing assays performed under 3D settings. Images are microphotographs of Caki-1 cells showing hole-closure of “tissue openings” generated with magnetic pattering as described in the materials and method. Cells were pretreated the same way with either Klotho (KL) or SB203580 and stimulated with EGF as described for the classical wound healing assay. **(D)** Plots of rate closure of holes for Caki-1 cells as a function of EGF exposure in the presence or absence of KL or SB203580.

Tumor cells undergo structural changes reminiscent of invasive species preceding cancer dissemination and are characterized by growth promoting activity. Growth factor-related effectors such as EGFRs and MAPKs play important activating function in this process [10–12, 14, 19–21]. Specifically, the pro-migratory function of p38 MAPK has been documented in certain cancers [22, 23] and the EGF-induced, cell migration on collagen type 1 mediated by α_2_β_1_ integrin requires p38 MAPK activation [24, 25]. Given that cRCC is a highly vascularized tumor expressing various angiogenic markers, growth-promoting events are undoubtedly important factors facilitating the tumor's progression. We have previously reported the inhibiting effect of secreted Klotho on p38 MAPK activation in kidney cell lines and in tissues of mouse brain [26–28]. Here we show that Klotho suppresses EGF-induced Caki-1 cell migration on collagen type 1 by inhibiting p38 MAPK activation. First, we followed the dynamics of EGF-induced p38 MAPK phosphorylation on Caki-1 cells, a cRCC cell model that expresses EGFR (Supplementary Figure 1). Serum-starved Caki-1 cells were treated with EGF (100ng/ml), and the relative level of p38 MAPK phosphorylation, as compared to total p38 MAPK expression, was detected by Western blot. As shown in Figure 1A, EGF induces p38 MAPK phosphorylation significantly at 5 min post-incubation with a reduction in phosphorylation intensity thereafter. Second, we tested whether soluble Klotho is capable of inhibiting the EGF-induced p38 MAPK phosphorylation. Cells were pretreated with either buffer only or 400pM Klotho for various incubation times and then stimulated with 100ng/ml EGF for 5 min. Klotho effectively inhibited p38 MAPK phosphorylation in a time-dependent manner (Figure 1B). As a positive control, SB203580, a specific p38 MAPK inhibitor, supplied at 1μM also inhibited EGF-induced p38 MAPK phosphorylation when preincubated with cells for 60 min (data not shown). Next, we determined the promigratory effect of EGF on Caki-1 cells and tested whether Klotho can impair the Caki-1 cell migration on collagen type 1. To do this, we conducted *in vitro* wound healing assays. EGF increased the rate of Caki-1 wound healing on collagen type 1 after 24 hours of treatment (Figure 2A). Preincubation of cells for 60 min with Klotho, by contrast, decreased the EGF-induced wound closure (Figure 2A). As a measure to account for p38 MAPK involvement in this process, we also employed SB203580 in the assay and observed profound inhibition of the wound closure (Figure 2A). Furthermore, we duplicated the wound healing assay in a 3D environment, which is supposed to mimic more closely the physiological situation *in vivo* [29]. Again, as shown in Figure 2C, an inhibitory pattern was observed similar to that of the conventional 2D assay. The quantified values of relative levels of the wound inhibition are summarized in Figure 2B&2D. These data suggest that the inhibitory effect of Klotho on EGF-induced Caki-1 cell migration on collagen type 1 is attributable partly to its inhibition of p38 MAPK activation. Future experiments in this direction, for example, will test whether Klotho overexpressing models, with suppressed p38 MAPK backgrounds will be necessary for EGF-induced cell migration, and vice versa.

### Klotho signaling interferes with cell adhesion and spreading, which are intermediary events preceding cell migration

**Figure 3:**
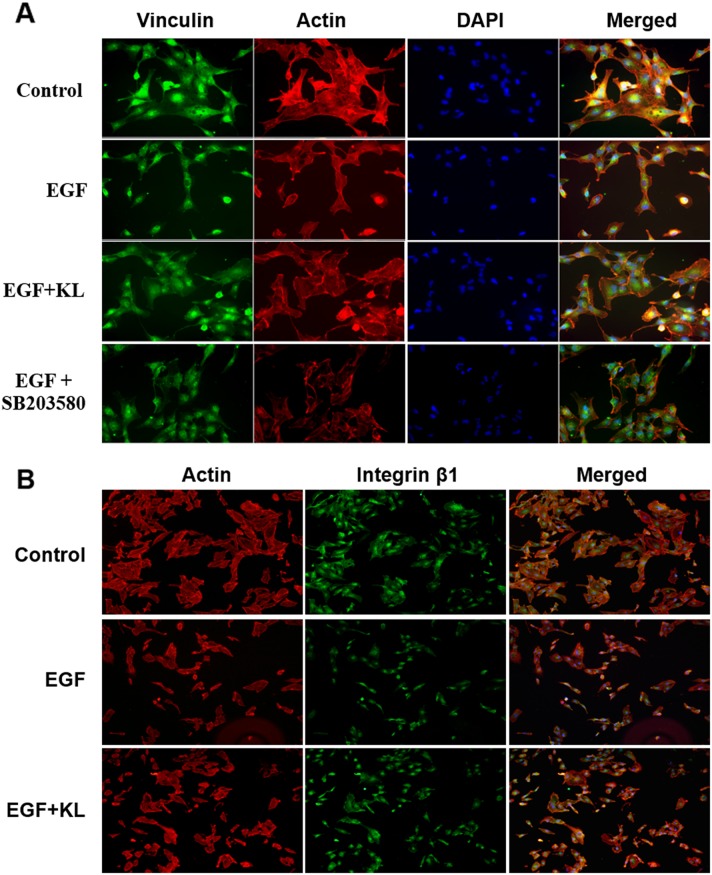
Klotho inhibits EGF-induced cell detachment from collagen type 1 coated adherent surface and affects the cell morphology change **(A)** Cells were grown on collagen type 1 coated dishes to 70% confluence and starved overnight. Starved cells were pretreated with Klotho or SB203580 for 60 min and then stimulated with 100ng/ml EGF for additional 15 min. Cells were then fixed and stained for F-actin (red) and vinculin (green). **(B)** Serum starved cells were trypsinized, pretreated with Klotho or buffer for 60 min, and then stimulated with EGF (100ng/ml) for 15 min; they were then seeded on collagen type 1coated glass chamber slides for 2 hours at 37°C/5% CO_2_. Cells were subsequently fixed and stained for F-actin (red), and integrin β1 (green).

Focal adhesion is involved in the key intermediate steps in morphological changes associated with cell migration. Vinculin, a cytoskeletal protein, is known to stabilize focal adhesions by regulating integrin clustering and the strength of the adhesion [10, 11]. By contrast, growth factors such as EGF, and MAPK destabilize focal adhesions [12, 15, 23, 30]. We hypothesized that Klotho regulated cell migration by stabilizing focal adhesions. We used immunofluorescent labeling for Vinculin (green) and F-actin (red) on Caki-1 cells, cultured on collagen type 1 to 70% confluence, to study how Klotho or the p38 MAPK inhibitor, SB203580, affects EGF-induced cell morphology and adhesion/detachment on collagen type 1, using these effects as surrogates for monitoring focal adhesion changes. As expected, EGF-only pretreatment caused profound morphological alterations, which triggered cell detachment from the substratum, mostly due to loss of focal adhesion complexes (Figure 3A). Some cells displayed rounded shapes, while others acquired spindle shapes similar to mesenchymal morphology. By contrast, pretreatment of cells with 400pM Klotho or 1μM SB203580 minimized the EGF-induced cell detachment and morphological changes (Figure 3A). In a related study, cells were first trypsinized, treated with EGF or Klotho as before, and further cultured for at least 2 hours to investigate the degree of cell attachment and/or morphology changes; we also investigated the involvement of integrins in this process by staining cells with integrin β1 antibody, and previously showcased the dependency of Caki-1 cell attachment to collagen type 1 through integrin α2β1 using both flow cytometry and real-time cell adhesion assays (Supplementary Figure 2). As shown in Figure 3B, EGF treatment delayed cell attachment and/or promoted cell detachment and pro-migratory morphological changes, while Klotho pretreated cells, on the other hand, inhibited these processes (see also Supplementary Figure 3 for the real-time cell adhesion assay). EGF-only treated cells were generally smaller and showed mesenchymal-like morphology and less focal adhesion containing integrin β1 compared to Klotho-pretreated cells. These studies suggest that Klotho actions antagonize pathways that promote focal adhesion disassembly and consequently may impair cell migration.

**Figure 4:**
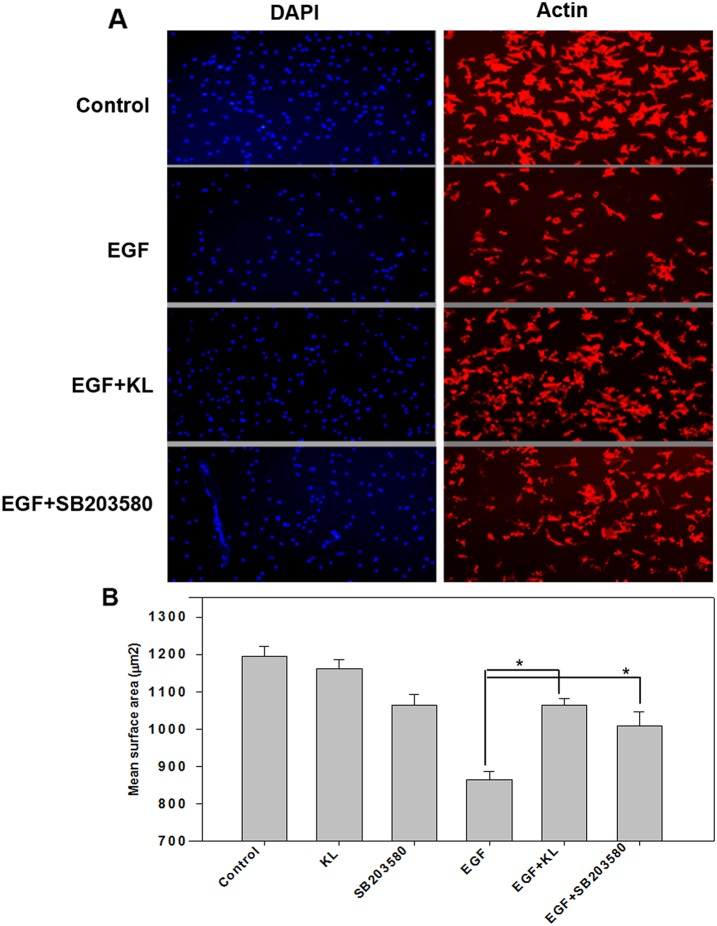
Klotho or p38 MAPK inhibitor SB203580 restrains EGF-induced Caki-1 cell surface reduction **(A)** Serum starved Caki-1 cells were trypsinized and pretreated with Klotho (KL), SB203580, or buffer alone for 60 min; this was followed by stimulation with EGF (100 ng/ml) for 15 min and seeded on collagen type 1-coated, microclear, 96-well plates for an additional 2 hours of culture at 37°C/5% CO_2_. Cells were immunostained for F-actin (red) or nuclear labeling (blue) and then imaged by fluorescent microscopy. **(B)** Summary of quantified mean surface area of cells measured using the ImageJ software as described in the text. The results represent means ± SEM of three independent experiments.

Cell spreading and migration are known to be regulated by growth factors and MAPKs [31, 32]. A previous study by Nakanishi's laboratory group [31] revealed that EGF antagonizes cell spreading on collagen type 1 in a MAPK-dependent manner. Here, we examined the effect of Klotho on Caki-1 cell spreading performed on collagen type 1-coated surfaces. Serum-starved cells were trypsinized, washed, and incubated in conditioned medium with either 400pM Klotho or 1μM SB203580 for 60 min; these cells were then treated with EGF for an additional 15 min and seeded on collagen type 1-coated, 96-well, clear microplates for at least 2 hours, at 37°C under 5% CO_2_. Cells were then fixed and stained for F-actin (red) and nuclear labeling (blue). Cell imaging was carried out using fluorescent microscopy and mean cell surface area was quantified using ImageJ software. As shown in Figure 4, EGF decreases mean cell surface area, and either Klotho or SB203580 inhibited the decrease. Quantified values for the various treatments are shown as histogram plots (Figure 4B). Taken together, these results, which describe the destabilization effect on cell morphology and attachment/detachment as a result of treatment with EGF, and the inhibition of that destabilization effect by Klotho or SB203580 thereof, are in agreement with previous reports indicating that EGF promotes cell migration by increasing focal adhesion disassembly and decreasing spreading through MAPK activation [12]. However, Klotho, which is known to inhibit p38 MAPK activity, and SB203580, a specific p38 MAPK chemical inhibitor, both antagonize the EGF-mediated effect. Collectively, our data suggest that EGF inhibits cell attachment and spreading and promotes cell detachment and cell migration by destabilizing focal adhesion complexes through a p38 MAPK-mediated pathway and that Klotho antagonizes these effects likely through inhibiting EGFR-p38 MAPK pathways.

### Klotho inhibits signaling proteins associated with the EGFR network

**Figure 5:**
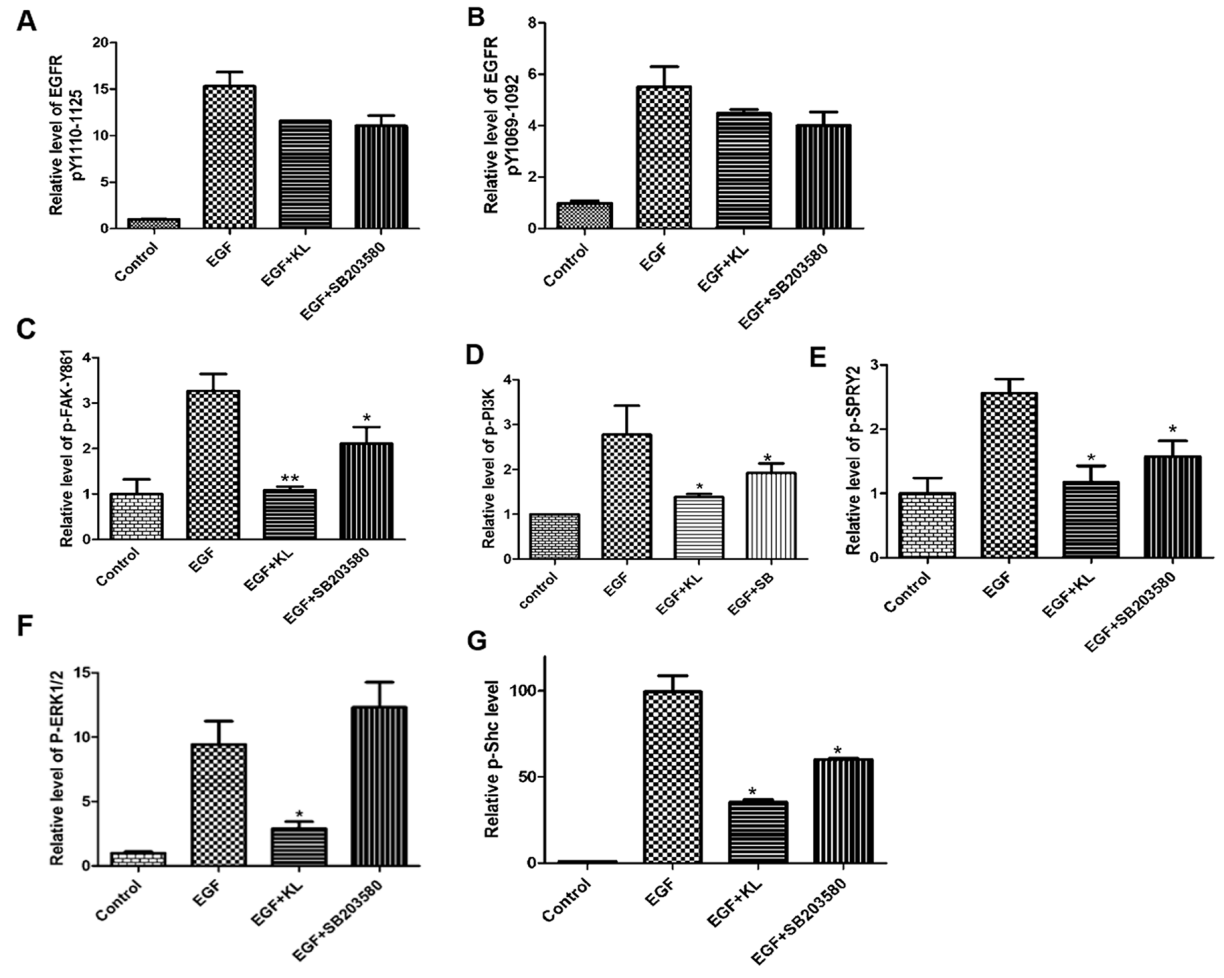
Klotho suppresses phosphorylation of signaling proteins activated by EGF stimulation of Caki-1 cells Cultured cells were either pretreated with Klotho (KL) (400pM), SB203580 (1μM), or buffer only for 60 min before been stimulated with EGF at determined time point. **(A-B)** Plots of phosphorylation levels of EGFR at two different site locations, **(C)** FAK, **(D)** PI3K, **(E)** SPRY2, **(F)** ERK1/2 and **(G)** Shc phosphorylation values were internally normalized utilizing the TAFII68 loading control.

To provide a more detailed molecular interpretation to our wound healing assays and morphology- based analyses, we looked into the regulation of signaling proteins associated with the EGFR network and whether Klotho had any inhibitory impact on these proteins. Focal adhesion contains many adaptor proteins such as vinculin, talin, and α-actinin, as well as tyrosine kinases such as FAK and Src that are activated upon extracellular matrix binding or growth factor stimulation [10–13]. Additionally, it has been shown that several proteins involved in EGFR signaling pathways can decrease focal adhesion stability and, consequently promote cell migration [12, 13, 31, 33]. We used a multiplexed immunoassay to assess the amounts of phosphorylated intracellular proteins involved in EGFR signaling in the presence or absence of secreted Klotho. Initially, we performed time dependent analyses of phosphorylation ranging from 0-120 min for selected markers and established optimal time ranges for subsequent EGF treatment (Supplementary Figure 4). As shown in Figure 5A&5B, Klotho or SB203580 pretreatment of Caki-1 cells for 60 min inhibited EGF-induced, EGFR phosphorylation at two different phosphorylated sites, though inhibition was relatively moderate. By contrast, Klotho, but not SB203580, profoundly inhibited the FAK-pY861 and ERK1/2 (Figure 5C&5F); PI3K, Shc, and SPRY2 phosphorylation was also highly inhibited by Klotho, whereas SB203580 showed intermediate inhibition (Figure 5D, 5E&5G). This suggests that Klotho works via the EGFR signaling network by inhibiting mostly downstream EGFR signaling proteins in concert with other reported growth factor receptors to suppress Caki-1 cell migration. The results also suggest that Klotho may inhibit EGF-induced ERK1/2 activation independent of p38 MAPK action.

### Klotho mRNA expression levels correlate with morphological characteristics in cRCC patients

**Figure 6:**
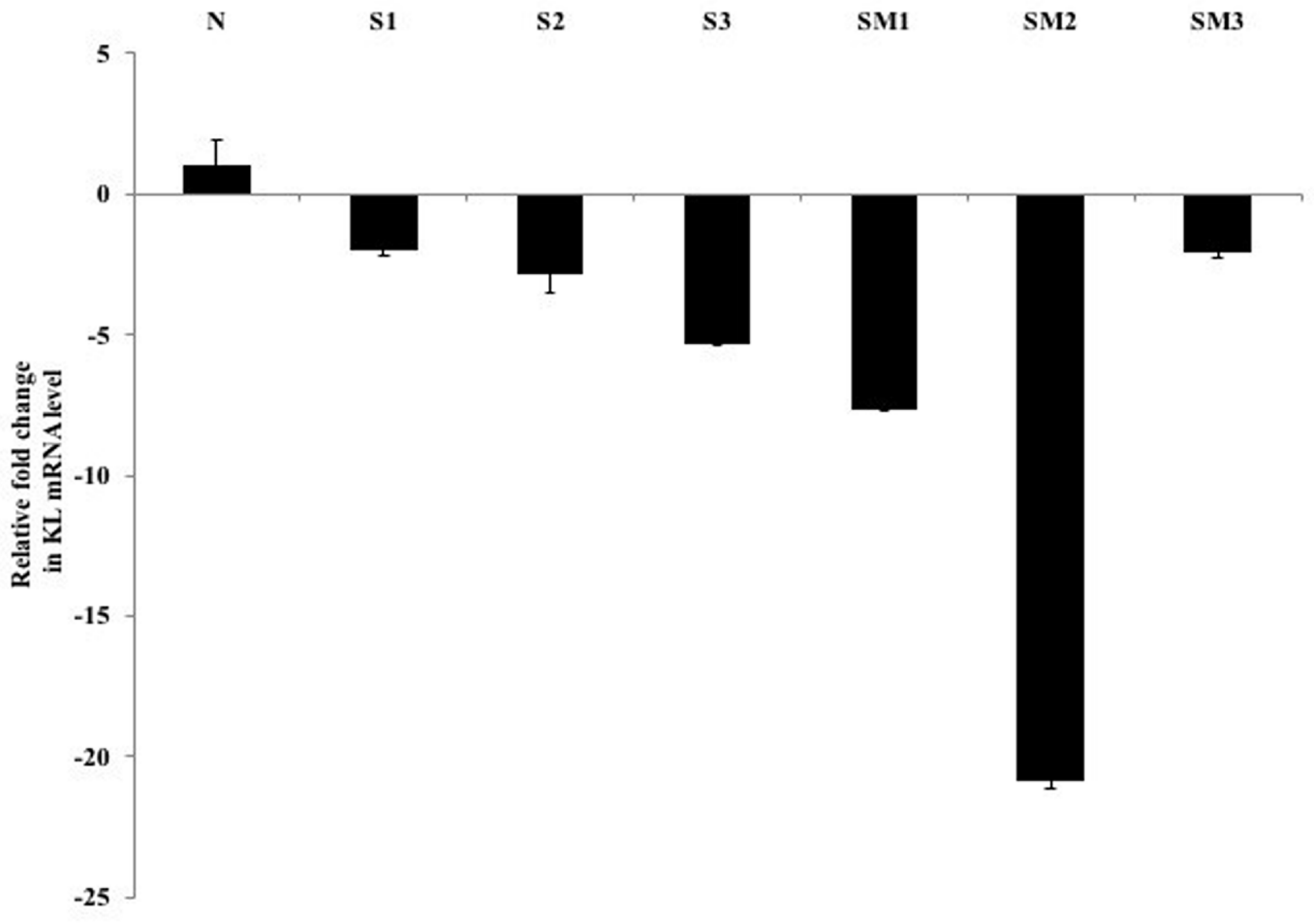
Fold reduction in Klotho mRNA expression in cRCC FFPE specimens Total RNA from microdissected FFPE tissues from three primary (S1-S3), three metastatic (SM1-SM3), and one normal control (non-cancer tissue) specimens were prepared as described in the text. Quantitative RT-PCR was performed to analyze Klotho and ACTB transcripts (tumor vs healthy) using a multiplexed, one-step Quantifast RT-PCR probe assay (Qiagen). The amount of mRNA encoding Klotho was assessed by relative quantification and the fold reduction expression changes were determined. All PCRs were performed in duplicate and deviations are shown as ± SEM.

The clinical relevance of Klotho-mediated inhibition of the EGF induced Caki-1 cell motility was verified in cRCC patients by expression analysis of six de-identified, cRCC FFPE tissue specimens (three primary tumors vs. three metastatic tumors) (Figure 6 and Table 1). In this retrospective study, we initially started with ten FFPE clinical specimens from which we obtained enough tissue from six for RNA extraction. We investigated endogenous Klotho mRNA levels in these patients to correlate Klotho's expression to the morphological features of tissue specimens. As Figure 6 and Table 1 indicate, there is a 2- to 20-fold reduction of Klotho mRNA expression in tumor isolates when normalized by ACTB transcripts in the primary and metastatic tumor groups versus a normal control. If Klotho mRNA levels correlate with protein expression, these results implicate Klotho as a physiologically relevant regulator molecule in cRCC pathogenesis and progression. The clinical data corroborate our earlier analysis of Klotho mRNA expression in Caki-1 cells relative to normal kidney that showed a greater than 3000-fold reduction (data not shown). Retrospectively, we also tested whether the differences in Klotho mRNA levels in the patients’ samples correlated with activation status and/or expression levels of some known cancer markers associated with cRCC progression. We found that whereas EGFR levels showed little or no difference between the primary tumor and metastatic RCC (Table 1), by contrast, c-Src protein was highly expressed in the metastatic tumors. Interestingly, E-Cadherin expression was essentially undetectable in most metastatic tumor specimens where Klotho expression is much decreased. Overall, our findings from the clinical samples establish a correlation between Klotho level and cRCC severity that match lower Klotho levels to worsening disease progression, although larger sample sizes will be needed to further validate the present discovery.

**Table 1:** Summary of normalized levels of Klotho mRNA expression from de-identified cRCC clinical cases with corresponding expression of selected cancer markers

Methods		qRT-PCR	SRM*^c^*		
Cases*^a^*	Biospy origin	KL mRNA n-fold change*^b^*	ECAD	c-Src	EGFR
S1	Kidney	-2.01±0.9	372±127	ND	268±68
S2	Kidney	-2.8±0.6	197±43	539±136	1496±194
S3	Kidney	-5.35±0.97	0	409±8.9	1812±69
SM1	Lung	-7.67±0.95	0	972±107	1244±23
SM2	Chest wall	-20.82±0.8	0	754±32	668±33
SM3	Lung	-2.07±0.88	443±4.5	ND	696±123

*^a^*De-identified FFPE slides from three cRCC primary tumor cases (SI-S3) and three metastatic tumors (SM1-SM3) were microdissected and the excised tissues were subjected to mRNA and protein analysis. *^b^*Quantitative RT-PCR was performed to analyze Klotho transcripts using a multiplexed, one-step, quantifast RT-PCR probe assay (Qiagen). The mRNA encoding Klotho was assessed by relative quantification and the fold reduction of expression was determined as referenced [20]. All PCRs were performed in duplicate and deviations are shown as ± SEM. *^c^*Selected reaction monitoring (SRM) mass spectrometry was used to obtain absolute quantitative analysis of EGFR, c-Src, and E-Cadherin (ECAD) levels. Analyses were performed in triplicate and deviations from the mean are shown. ND, not determined.

### Concluding remarks

Our data support the growing evidence that Klotho participates in cellular signaling activities that negatively impact cancer cell growth, motility and invasion [3, 4, 6–9, 34–36]. This activity is demonstrated in our wound healing assays and, also, by Klotho's ability to interfere with cell adhesion and/or detachment. We also showed that suppression EGFR-MAPK pathway by Klotho induced cells to adopt mesenchymal like (spindle-shaped) morphology. These effects appear to be borne from an inhibition of p38 MAPK phosphorylation and a suppression of the EGFR signaling complex and is coupled to the downregulation of selected markers known to associate with EMT. Moreover, expression analysis of FFPE sections from cRCC clinical specimens correlate decreased Klotho expression to increased levels of some selected EMT markers. These developments place Klotho centrally to mechanisms that regulate cRCC cell motility in response to physiological exposure. We also demonstrate that, part of Klotho function is stabilizing focal adhesion complexes, and therefore fostering cell attachment and spreading. If locally secreted Klotho prevails within the tumor microenviroment, either Klotho would physically interfere with recruitment of integrins and their allied effectors to the focal adhesion complexes via the EGFR network, thus minimizing their effectiveness, or, Klotho actions indirectly send signals that interfere with collagen degradation and/or redeposition that are also mechanisms of tumor migration [17]. Whereas we have yet to independently support some of these proposed mechanisms experimentally, literature abounds with studies on Klotho's inhibitory role on growth factor receptor signaling with respect to cell migration. Moreover, the relatively lower Klotho mRNA levels observed in our cRCC FFPE clinical specimens, and their correlation to higher/lower expression levels of EMT markers, suggest a relevance of Klotho expression to cRCC pathogenesis. A larger collection of clinical samples is yet in need to confirm this correlation, and consequently Klotho expression level can potentially be used as a biomarker to monitor disease progression.

The EGFR network has long been a molecular target of intervention in diverse cancers, and EGFR-targeted cancer drugs continue to be used in clinics. New compounds are being developed for precision therapy based on our new understanding of the receptor signaling mechanisms. This network understanding revolves round Ras/Raf/MAPKs or PI3K/Akt pathways, where downstream events lead to nuclear translocations of signaling molecules (MAPKs/Akt), and subsequent transcriptional activations, or FAK activation that are relevant to cell proliferation and growth, apoptosis, invasion, and adhesion/migration. In addition, a deeper understanding of EGFR signaling via Src kinases, PLCγ/PKC, and STATs has demonstrated a link of these pathways to tumor progression and survival. More recently, a refined oncogenic role of EGFR has emerged that sheds further insight into mechanisms of the receptor activation, including the discovery of somatic mutations, receptor gene amplification, gene deletions, novel or aberrant alternative splice variants, and nuclear localization [37]. Whereas somatic mutations in the *EGFR* gene and/or receptor amplification in certain cancers such as lung, breast, ovarian, and glioblastomas have been linked to a boosted response to the anti-EGFR tyrosine kinase inhibitors (TKIs) gefitinib and erlotinib in some cases [38–41], a high nuclear EGFR presence is an indication of poor response to therapy and increased disease severity [42–46]. Unfortunately, the same cannot be said of cRCC tumors, where, to date, only a subset of cRCC patients are known to respond to TKIs. It should be noted that sequence analysis in a cohort of RCC tumors conducted in the past found no somatic mutations in *EGFR*, *KRAS*, or *BRAF* that appear to influence any tumor response to TKIs [47]. This suggests that innovations in pathway analyses in cRCC tumors based on EGFR signaling are urgently needed. Judging from Klotho's ubiquitous role in cell signaling, this study provides another linkage for pathway exploration in cRCC tumors. Thus, a potential role of Klotho as a predictive biomarker for cRCC progression cannot be overemphasized.

## MATERIALS AND METHODS

### Antibodies and chemicals

RPMI 1640, fetal bovine serum, trypsin-EDTA, penicillin and streptomycin combination, cell dissociation solution, enhanced chemiluminescence detection reagents, and anti-human rabbit monoclonal vinculin antibody were all purchased from Life Technologies (Carlsbad, CA). Recombinant human EGF was from PeproTech (Rocky Hill, NJ). Human recombinant secreted Klotho was purchased from R&D Systems (Minneapolis, MN). FITC-conjugated goat anti-mouse and TRITC-conjugated goat anti-rat were purchased from Jackson Immunoresearch (West Grove, PA). Dylight™ goat anti-rabbit IgG and anti-human mouse monoclonal integrin β1 antibody were obtained from Thermo Scientific (Waltham, MA). HRP-conjugated goat anti-rabbit IgG was from Bio-Rad (Hercules, CA). Mouse anti-human monoclonal and rabbit anti-human polyclonal EGFR antibodies were products of Abcam (Cambridge, MA). Rabbit anti-human polyclonal active p38 MAPK antibody and SB203580 were purchased from Promega (Madison, WI). Rabbit anti-human polyclonal p38 MAPK antibody was acquired from Calbiochem. Mouse anti-human monoclonal α2β1 antibody (clone BHA2.1) was purchased from Chemicon (San Diego, CA). Collagen type I was procured from Upstate Biotechnology (Waltham, MA).

### Cell culture

Human Caki-1 cells originated from a cRCC patient (ATCC HTB-46), were cultured in RPMI 1640 medium supplemented with 10% fetal bovine serum and penicillin/streptomycin at 37°C in a humidified atmosphere containing 5% CO_2_. Cells were grown to 80% confluence and then serum-starved overnight prior to treatment with recombinant factors and/or medium components.

### 2D Wound healing assay

Caki-1 cells were seeded on a collagen type 1-coated, 6-well plate and grown overnight to 80% confluence. After overnight serum starvation, the monolayers were scratched with a 200-μL pipette tip to create a wound and then washed twice with serum-free RPMI 1640 to remove floating cells. The culture wound was photographed at time 0 and 24 h later, and the rate of closure was assessed.

### 3D Wound healing assay

For 3D cell cultures, surface attached cells (grown to 80% confluence) were treated with 1ml of Nanoshuttle magnetic beads solution (n3D Biosciences, Houston, TX) per 1cm^2^ of surface area available for cell culture and incubated overnight. Treated cells were detached and subcultured into non–tissue culture–treated 24-well plate dishes overnight. After serum starvation, the cells were shredded; these were patterned in 96-well plates. Then, using ring-shaped magnet drivers, holes were made within the tissues.

### Immunoblotting

Immunoblotting was performed to measure p38 MAPK activation levels by protein phosphorylation detection in the presence or absence of EGF. Cells were lysed in RIPA buffer, and then the samples were heated to 95°C for 5 min, separated on a 4%–12% Bis-Tris NuPAGE gel (Life Technologies) and transferred to nitrocellulose for immunoblotting. Immunoblots were incubated with primary, then secondary antibodies and bound antibody levels were detected with the Novex^R^ ECL Western blotting detection reagents (Life Technologies).

### Multiplexed immunoassays

Cell lysates were processed according to the MILLIPLEX Map EpiQuant sample preparation kit (Millipore, Burlington, MA); then, multiplex immunoassays were performed in a 96-well plate using the Epiquant™ EGFR pathway magnetic bead panel as instructed (Millipore).

### Real-time cell adhesion assay

The xCELLigence system was used as instructed (ACEA Biosciences, San Diego, CA). Briefly, collagen type 1–coated ACEA 96-well E-plates were used for real-time measurement of cell adhesion using an xCELLigence RTCA SP system. First, a standard background measurement was performed using 50μl of cultivation media. Starved Caki-1 cells were trypsinized, quantified, treated with EGF and/or Klotho, and loaded into the wells at 10,000 cells/well. The cells were monitored continually every 3 min for a total of 3 hours. Data are presented in relation to the cell index. Cell index (CI) is an unitless parameter that corresponds to cell status and is derived from the measured relative change in electrical impedance that occurs in the presence and absence of attached cells in the wells.

### Immunofluorescent microscopy

Caki-1 cells were seeded on collagen type 1 and incubated at 37°C in a humidified atmosphere containing 5% CO_2_ for appropriate times based on the experiment. Cells were washed twice in phosphate-buffered saline (PBS) and fixed for 30 min in 4% paraformaldehyde. Slides were then washed twice in PBS and once in PBS containing 0.05% Triton X-100 for 5 min. Subsequently, the cells were incubated in 3% bovine serum albumin (BSA) in PBS for 1 hour. Both the actin cytoskeleton and integrin β1 were stained using specific fluorescence-tagged probes (rhodamine-conjugated phalloidin for F-actin, DAPI for nucleus and FITC-conjugated Ab for integrin β1) and mounted with prolong gold anti-fade reagent (Life Technologies).

### Cell spreading assay

Cell spreading was performed in collagen type 1-coated, 96-well plates. Non-specific binding was blocked with heat-denatured BSA. Starved cells were detached and resuspended in medium without serum and treated with stimuli and/or inhibitors; they were then loaded into the coated wells and incubated for 2 hours. At the end of the incubation period, cells were fixed with formaldehyde, solubilized with Triton x-100, and then they were stained with F-actin and DAPI and imaged by fluorescence microscopy. Total cell/well areas occupied by cells and cell numbers were measured using ImageJ software and by dividing total cell surface area by cell numbers. The mean surface area occupied per cell was then calculated.

### Sectioning, staining, and laser microdissection

Sectioning of the paraffin-embedded samples at 5μm thickness was performed on a microtome with a sterile disposable blade. Sample sections were mounted on UV-treated slides for laser microdissection (LMD). The slides were dried in an incubator at 37°C for 3 hours. Hematoxylin and eosin staining was performed as discussed elsewhere [48]. Briefly, the slides were rehydrated, stained, and dehydrated, followed by drying for 1 hour at room temperature. Then the slides were covered by 2% Magic Mount Solution (Jung Woo F&B, Seoul, South Korea). We used the LMD ION II system (Jung Woo F&B) which is an upright microscope and uses an infrared laser to dissect. Target cells were preselected on a monitor with a drawing pen, and then the laser beam was moved robotically along the pre-selected path and target cells were excised from the section. The dissected cells then fell into caps under gravity. Dissected samples were viewed within the caps for authentication.

### RNA extraction and quantitative RT-PCR

Total RNA from FFPE microdissected tissues were extracted using the RNeasy FFPE Kit (Qiagen, Germantown, MD) as instructed. Briefly, the captured cells were resuspended in PKD buffer containing 10μl proteinase K (Qiagen), followed by incubation at 80°C for 15 min to reverse formalin cross-linking of the released nucleic acids. The lysates were then thoroughly mixed with red blood cell buffer (Qiagen), pipetted into a gDNA Eliminator spin column (Qiagen), and centrifuged. The samples were then transferred into an RNeasy MinElute spin column (Qiagen) and placed in a 2-ml collection tube, where the total RNA binds to the membranes and contaminants are efficiently cleared. Finally, RNA was eluted in nuclease free water. The quality and quantity of isolated total RNA were assessed on a 2100 BioAnalyzer using the RNA 6000 Pico Assay as instructed (Agilent Technologies, Austin, TX). The resultant electropherograms were used to determine RNA integrity and concentration. Quantitative RT-PCR was performed for the analysis of Klotho transcripts using the Rotor-Gene Q system (Qiagen). We used the multiplexed, one-step quantifast RT-PCR probe assay (Qiagen) for quantification of Klotho and ACTB transcripts within RNA extracted from tumor and healthy tissues. All PCRs were performed in duplicate. No-template controls (NTCs) were included in each run. The amount of mRNA encoding Klotho was assessed by relative quantification and the fold-reduction expression changes were determined as described elsewhere [49].

### Selected reaction monitoring mass spectrometry

Absolute quantitative analysis of IGF-1R, c-Met, EGFR, BIM, c-Src, actin, tubulin, and E-Cadherin and relative analysis of N-Cadherin, on patient tissue samples were performed using selected reaction monitoring (SRM) mass spectrometry (MS) assays developed by Expression Pathology Inc. (EPI, Rockville, MD). The study material consisted of two FFPE sections from 14 formalin-fixed kidney tissue samples which were cut and placed on DIRECTOR^®^ microdissection slides according to EPI preparation instructions. Ten (10) of 14 samples held enough material for the analysis defined by EPI inspection. Briefly, tissue sections on DIRECTOR^®^ slides were deparaffinized and stained with hematoxylin to prepare them for microdissection following standard operating procedures. Tumor cells were procured from the tissue sections by LMD utilizing the Leica LMD6000 tissue microdissection instrument employing standard methods to collect approximately 45,000 cells per sample. The number of cells was estimated by extrapolating the total area of microdissected tissue. The total area is calculated as a function of the LMD6000 instrument. Expression Pathology's Liquid Tissue^®^ MS Protein Prep kit was used following the manufacturer's instructions to prepare protein lysates from each sample. This kit uses proprietary reagents to solubilize and digest the proteins for subsequent MS analysis. Protein lysates were made from microdissected specimens from each tissue sample for quantitative purposes. The total protein yield recovered from each sample was determined in a Micro BCA Assay. An aliquot of each sample preparation containing 2.0μg of protein was prepared and diluted to 40μl with 0.1% formic acid, and 4μl of heavy standard peptide mixture (5fmol/ml) was added to each sample. Following a centrifugation step at 10,000×g for 10 minutes, 45μl of the supernatants were placed in the system's auto sampler. A 10μl of each sample containing 0.5μg total protein and 5fmol of internal standard peptide was injected into the MS system at a flow rate of 5μl/minute and eluted at 0.8μl/minute. All study samples were analyzed in triplicate. Peptides were eluted from the reverse-phase column for 30 minutes. The MS was run in the SRM mode with an injection of 0.5μg total protein, a dwell time of 10ms, Q1 FWHM of 0.2 and Q3 FWHM of 0.7. SRM data was inspected using Xcalibur 2.1 software (Thermo Scientific) and analyzed using Pinpoint software (Thermo Scientific). Individual data points and MS peaks acquired from each sample were confirmed by manual review of the data directly in the Xcalibur program. Correct peaks were confirmed by direct visualization of product ions ratios. Quantitative measurement of each analyte was determined by peak area comparison between the spiked internal standard peptides and the native peptides by calculating the ratio of the endogenous analyte to the signal of the heavy-labeled isotope, multiplied by the amount of heavy isotope spiked in. For each sample, the arithmetic mean, standard deviation, and %CV (Mean/SD^*^100) were calculated.

### Statistical analysis

Analyses were routinely done using 2-tailed *t* tests, where needed. Data were presented as means ± SEM. Tests were performed for Klotho vs EGF to score the effect of Klotho inhibition of signaling proteins activated by EGF stimulation at a significance level of 0.05. Where applied, (^*^) represents statistically significant disparities between the entities tested.

## SUPPLEMENTARY MATERIALS AND FIGURES



## Abbreviations

cRCC: clear-cell renal cell carcinoma
KL: Klotho
TKI: tyrosine kinase inhibitor
SRM: selected reaction monitoring
MS: mass spectrometry
LMD: laser microdissection
ACTB: beta-actin
FWHM: full width at half maximum
CI: cell index
ND: not determined
