# Supplementary material for: Klotho inhibits EGF-induced cell migration in Caki-1 cells through inactivation of EGFR and p38 MAPK signaling pathways
Source: Oncotarget. 2018 Jun 1;9(42):26737–50. doi: 10.18632/oncotarget.25481 (PMC6003560; doi:10.18632/oncotarget.25481)
Supplement: Supplementary file 1 [file oncotarget-09-26737-s001.pdf]

# Klotho inhibits EGF-induced cell migration in Caki-1 cells through inactivation of EGFR and p38 MAPK signaling pathways

## SUPPLEMENTARY MATERIALS

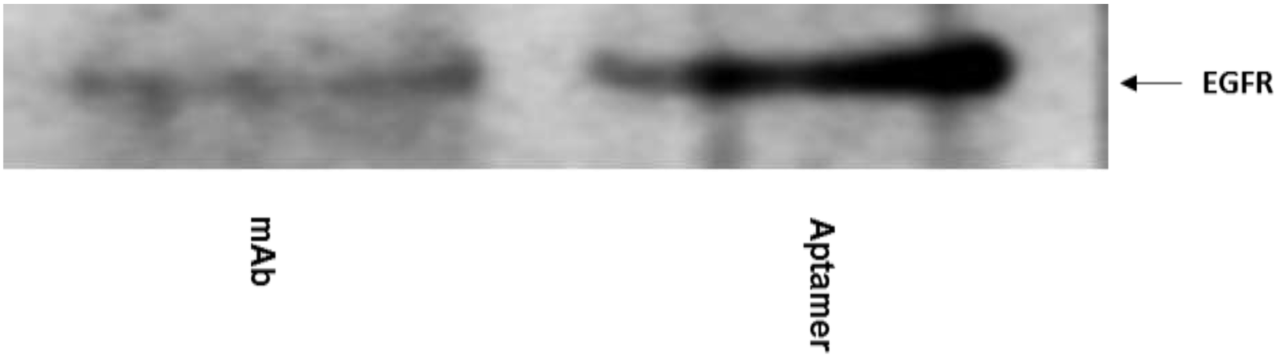


**Supplementary Figure 1: Detection of EGFR expression in Caki-1 cells using antibody- and aptamer-based chemistries.**

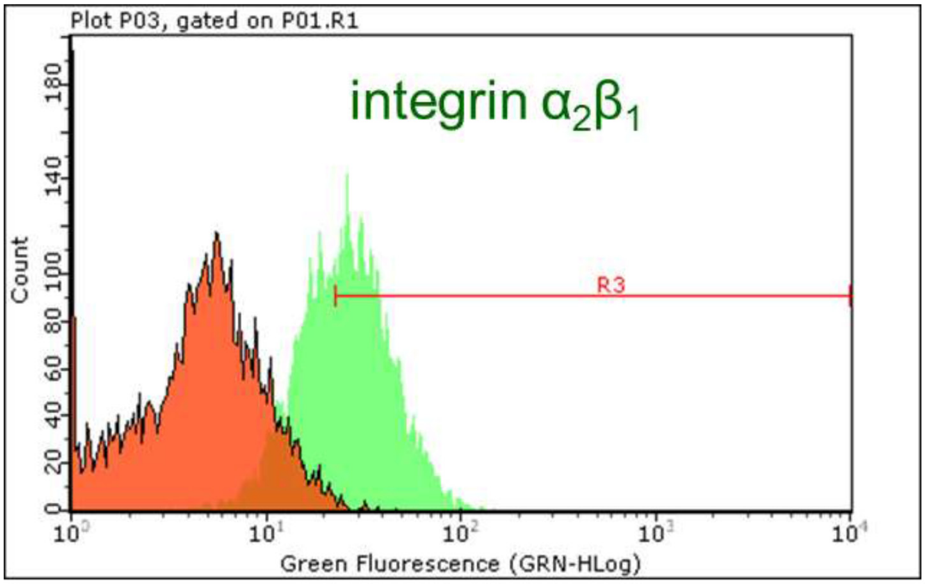


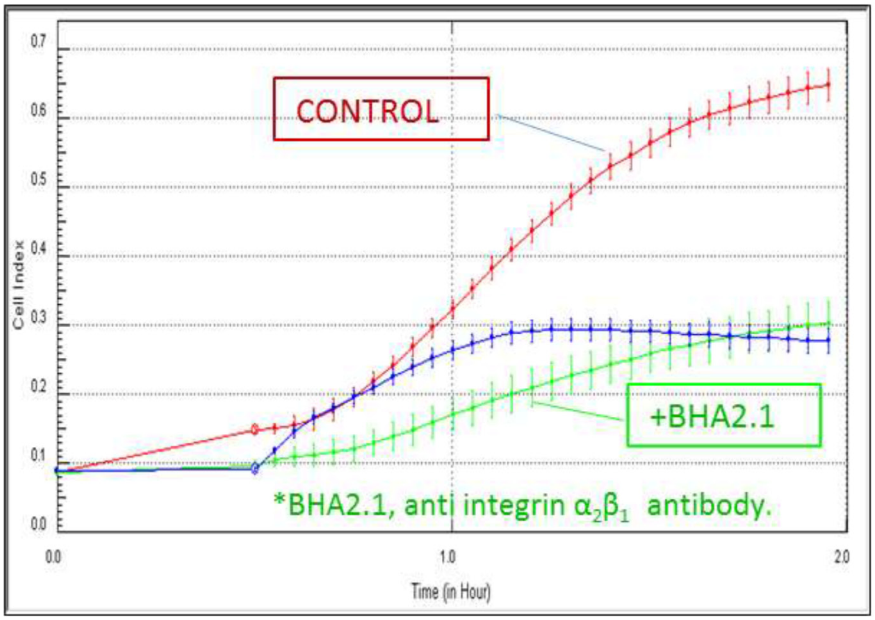


**Supplementary Figure 2: Attachment of Caki-1 cells to collagen type 1-coated matrix is integrin α2β1-dependent.**

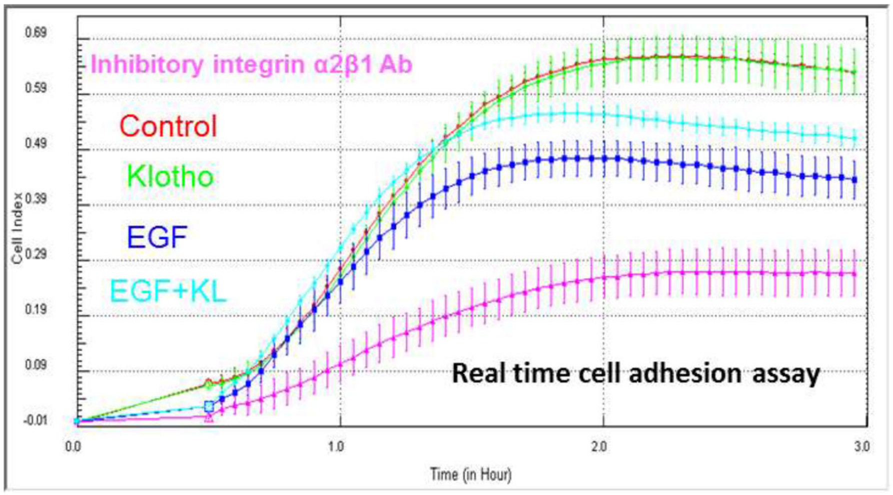


**Supplementary Figure 3: Real-time, cell-adhesion assay demonstration using the xCELLigence RTCA SP system.**

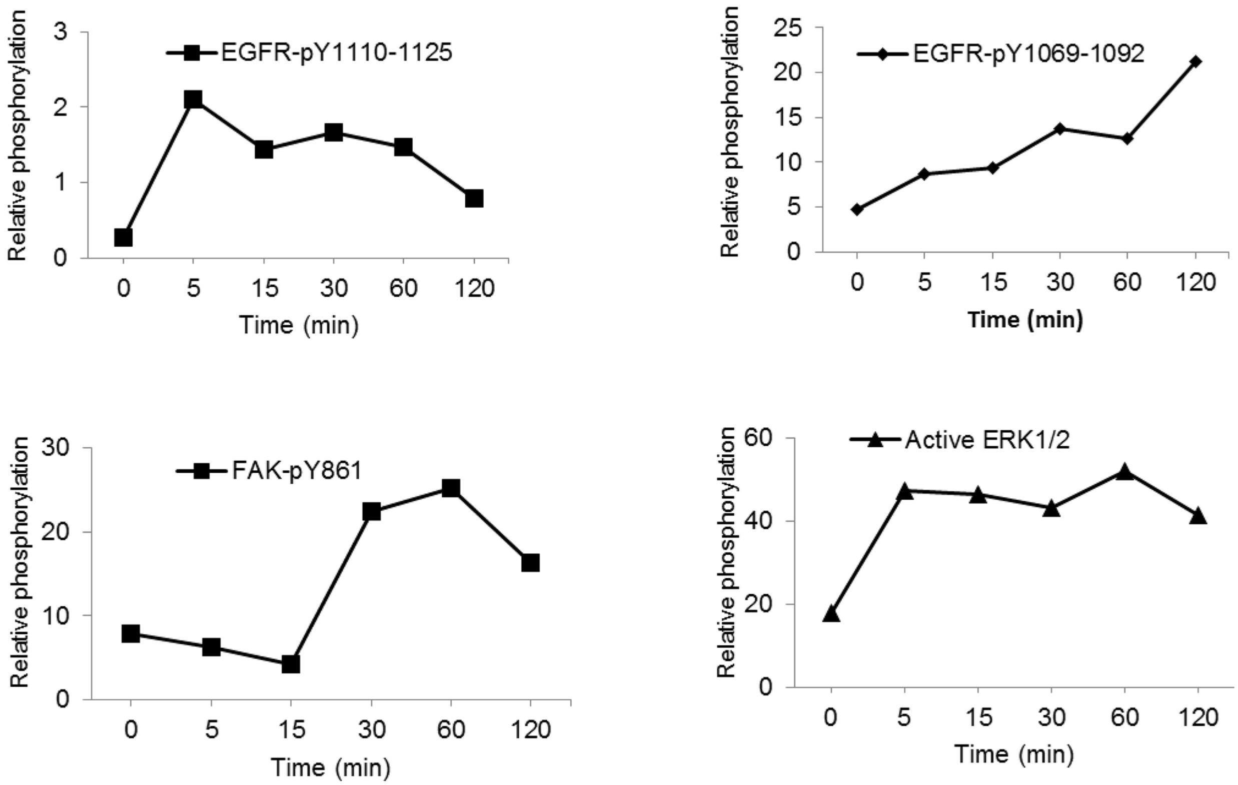


**Supplementary Figure 4: Plots of time-dependent phosphorylation of signaling proteins from EGF-stimulated Caki-1 cells.**
